# Peer Review for “Improved Alzheimer Disease Diagnosis With a Machine Learning Approach and Neuroimaging: Case Study Development”

**DOI:** 10.2196/73130

**Published:** 2025-04-21

**Authors:** Anonymous

**Keywords:** Alzheimer disease, computer-aided diagnosis system, machine learning, principal component analysis, linear discriminant analysis, t-distributed stochastic neighbor embedding, feedforward neural network, vision transformer architecture, support vector machines, magnetic resonance imaging, positron emission tomography imaging, Open Access Series of Imaging Studies, Alzheimer's Disease Neuroimaging Initiative, OASIS, ADNI

*This is a peer-review report for “Improved Alzheimer Disease Diagnosis With a Machine Learning Approach and Neuroimaging: Case Study Development.”*

## Round 1 Review

### General Comments

The paper [1] discusses the development of a machine learning–based computer-aided diagnosis system for the detection and classification of Alzheimer disease. The system uses brain magnetic resonance imaging and positron emission tomography images from the Open Access Series of Imaging Studies database, applying principal component analysis for feature extraction and using support vector machines (SVMs) and artificial neural networks (ANNs) as classifiers. Although the proposed model shows relatively good performance, the paper should focus on justifying the novelty of the method and providing more details in the results.

### Specific Comments

#### Major Comments

1. The paper lacks a clear discussion on how the proposed method substantially advances the state of the art. While it combines principal component analysis with SVM and ANN, similar combinations have been explored in prior research. The authors should clearly write about how their work is novel and the specific contributions made beyond existing studies.
2. The paper does not provide sufficient details on the hyperparameter tuning process for both SVM and ANN models. The review suggests that the author include these additional details in an appendix.
3. The evaluation primarily focuses on accuracy, sensitivity, and specificity. However, other metrics like precision, *F*_1_-score, and area under the receiver operating characteristic curve could provide a more comprehensive assessment of the model’s performance. The authors could consider adding additional metrics for evaluation.
4. In Figure 2, the size of the box on the left and right are different (square vs rectangle). Is there a specific reason the author made this design choice?

#### Minor Comments

1. The paper’s organization can be improved. Some sections, like the methodological explanation of principal component analysis, are overly detailed, while others, like the description of SVM and ANN, are relatively brief. Please consider balancing the sections.
2. The Related Work section is somewhat sparse and does not sufficiently cover recent advances in the field. Please consider adding more recent studies.

## Abbreviations

ANN: artificial neural network
SVM: support vector machine
